# When Care Meets Conflict: Attitudes Toward Restraints Among Psychiatric Trainees and Early Career Psychiatrists

**DOI:** 10.1192/j.eurpsy.2026.10752

**Published:** 2026-07-31

**Authors:** A. Olteanu, I. Sungur, D. Zani, S. M. Nouri, F. Monshizadeh Tehrani

**Affiliations:** 1 Clinical Hospital of Psychiatry “Prof. Dr. Alexandru Obregia”, Bucharest, Romania; 2 Department of Psychiatry, Ege University School of Medicine, Izmir, Türkiye; 3 Center for Forensic Psychiatry, Psychiatric Services Grisons, Cazis; 4 Institute of Biomedical Ethics and History of Medicine, University of Zurich, Zurich, Switzerland; 5 Zanjan University of Medical Sciences, Zanjan, Iran, Islamic Republic Of; 6 Child and Adolescent Mental Health Center, Mental Health Services in the Capital Region of Denmark, Copenhagen, Denmark

## Abstract

**Introduction:**

Working in psychiatry is highly rewarding but can also carry occupational risks. Throughout their career, trainees and early career psychiatrists (ECPs) may be exposed to or witness different forms of patient aggression. Such direct or indirect exposures may lead to post traumatic stress symptoms (PTSS), shape clinicians’ perception of risk, and alter their attitudes toward the use of restraints (ATR) as a means of preventing harm. However, little is known about how direct or indirect exposure to patient aggression is associated with the development of PTSS and changes in ATR among trainees and ECPs.

**Objectives:**

The aim of this study is to report the frequency of self-reported recent direct and indirect exposure to patient aggression among European trainees and ECPs and to explore its association with PTSS and ATR.

**Methods:**

A cross-sectional, anonymous online survey was conducted among European psychiatric trainees and ECPs. The survey included two newly developed scales: (a) PTSS (α = 0.926) and (b) ATR (α = 0.805). The survey was reviewed for clarity and fluency, and reliability was confirmed with Cronbach’s α. Participants reported frequency of direct and indirect exposure to patient aggression encompassing verbal threats, physical threats, assaults, and sexual aggression over the past 12 months on a five-point Likert scale. Scores were summed for analysis. Data were analysed using SPSS (v. 27). Normality was examined by Shapiro–Wilk, and histogram inspection. Given the non-normal distribution, associations between main variables were examined using Spearman’s correlation. Statistical significance was set at p < 0.05.

**Results:**

Of the 206 participants from 24 European countries, 74% were trainees and 26% ECPs. The mean age was 30.5 years (SD = 5.4, range 24–56). All participants reported at least one direct or indirect exposure to patient aggression within the past 12 months.

Spearman’s correlation indicated a small but significant positive association between ATR and frequency of both recent direct (rs = 0.196, p = 0.005) and indirect aggression (rs = 0.145, p = 0.038). Concurrently, a moderate and significant positive correlation was found between frequency of recent indirect exposure and PTSS (rs = 0.32, p < 0.001).

**Conclusions:**

This study is the first multinational investigation to explore the association between frequency of recent exposure to patient aggression and PTSS and ATR among psychiatry trainees and ECPs. The high prevalence of recent direct and indirect exposure to patient aggression and the significant correlation of their frequence to both PTSS and clinicians’ favourable ATR indicate the need for targeted training and institutional policies to promote safety, both to ensure clinicians’ mental well-being and to reduce the use of restraints.

**Disclosure of Interest:**

None Declared

